# Increasing and Decreasing Alcohol Use Trajectories Among Older Women in the U.S. Across a 10-Year Interval

**DOI:** 10.3390/ijerph8083263

**Published:** 2011-08-05

**Authors:** Janet Kay Bobo, April A. Greek

**Affiliations:** Centers for Public Health Research and Evaluation, Battelle Memorial Institute, 1100 Dexter Ave, North, Suite 400, Seattle, WA 98109, USA; E-Mail: greeka@battelle.org

**Keywords:** alcohol use, trajectories, older women, risk factors

## Abstract

Older women who routinely drink alcohol may experience health benefits, but they are also at risk for adverse effects. Despite the importance of their drinking patterns, few studies have analyzed longitudinal data on changes in drinking among community-based samples of women ages 50 and older. Reported here are findings from a semi-parametric group-based model that used data from 4,439 randomly sampled U.S. women who enrolled in the Health and Retirement Study (HRS) and completed ≥ 3 biannual alcohol assessments during 1998–2008. The best-fitting model based on the drinks per day data had four trajectories labeled as “Increasing Drinkers” (5.3% of sample), “Decreasing Drinkers” (5.9%), “Stable Drinkers” (24.2%), and “Non/Infrequent Drinkers” (64.6%). Using group assignments generated by the trajectory model, one adjusted logistic regression analysis contrasted the groups with low alcohol intake in 1998 (Increasing Drinkers and Non/Infrequent Drinkers). In this model, baseline education, physical activity, cigarette smoking, and binge drinking were significant factors. Another analysis compared the groups with higher intake in 1998 (Decreasing Drinkers *versus* Stable Drinkers). In this comparison, baseline depression, cigarette smoking, binge drinking, and retirement status were significant. Findings underscore the need to periodically counsel all older women on the risks and benefits of alcohol use.

## 1. Introduction

### 1.1. Public Health and Alcohol Use Patterns among Older Women

All possible levels of drinking, including abstinence, can influence the health and well-being of women ages 50 and older. Moderate drinking, which the United States (U.S.) Department of Agriculture in their *Dietary Guidelines for Americans 2010* has defined as up to one drink per day for women and no more seven drinks per week [1], offers important cardiovascular [2] and cognitive [3–5] benefits, especially among postmenopausal women [6] and may lower all-cause mortality [7,8]. However, many health care providers are reluctant to advise their older patients to drink, because even moderate intake can encourage weight gain [1], interact adversely with many medications commonly prescribed to the elderly [9], and lead to problem drinking. Alcohol abuse is a well-established risk factor for many of the leading causes of early mortality among women [10,11]. For all these reasons, public health practice needs reliable data on alcohol use patterns among older women.

The analyses reported here were undertaken to accomplish three objectives. First, the study was designed to identify common drinking trajectories in a randomly selected sample of women ages 50–65 at baseline who were followed for 10 years. This objective assumed the existence of homogeneous alcohol use subsets within a heterogeneous sample. The second objective was to obtain weighted estimates of the percentages of women associated with each of the observed trajectories in a representative sample. In a prior study, we found that nearly 9% of a randomly selected sample of older women steadily increased their number of drinks per drinking day over 8 years of follow-up [12]. Finally, we sought to identify demographic, health, and substance use characteristics at baseline that may influence a woman’s risk of following either a trajectory characterized by increasing alcohol use that could place her at risk for becoming a problem drinker or a trajectory of decreasing use that would deprive her of the potential health benefits of moderate drinking.

Gender-specific analyses were conducted for this study, because cross-sectional data have amply demonstrated that older men and women drink differently [13–15]. Although some investigators combine male and female respondents in a single dataset and then include gender as a term in their multivariate models, this approach may obscure important information. Previously, we reported on changes in drinking behaviors over time (alcohol use trajectories) among 3,105 older men enrolled in the Health and Retirement Study (HRS) [16].

### 1.2. Identifying Homogenous Subgroups within a Heterogeneous Sample

A variety of statistical tools have been developed to study intra-individual change over time (e.g., latent class analysis, growth curve modeling, mixture models, and semi-parametric group-based models) [17]. In contrast to more traditional variable-centered analyses, these person-centered methods require longitudinal data and emphasize characteristics associated with change over time (e.g., rate of change, degree of change, starting points). Some methods assume a single underlying latent pattern, but others avoid a priori assumptions about the number and nature of distinct latent classes or trajectory groups within a defined population.

Social scientists have used person-centered methods to study a wide variety of topics related to behavior change over time including alcohol use after exposure to an urban disaster [18], drinking during adolescence [19], drinking among college students [20], development of psychiatric trajectories in adults [21], and tobacco use during adolescence and early adulthood [22]. To identify the most common types of alcohol use trajectories followed by older women over a 10-year interval, we used extant longitudinal data from a representative sample in the U.S. to construct semi-parametric group-based models using methods developed by Nagin and colleagues [23–27].

## 2. Experimental Section

### 2.1. Study Participants

For this investigation, we obtained Health and Retirement Study (HRS) files available at http://hrsonline.isr.umich.edu/ for the 1931–1941 and 1942–1947 birth cohorts. Both cohorts were identified via national area probability samples of U.S. households with over-sampling of Blacks, Hispanics, and residents of Florida [28]. Birth-cohort eligible spouses and co-resident partners of cohort members were also enrolled and assigned sampling weights.

Members of the older birth cohort were enrolled in 1992 during an in-person interview and then invited to complete biannual telephone interviews from 1994 on. The younger cohort was enrolled in 1998, interviewed in-person with the same items administered to the older cohort in that year, and then merged with the older cohort for all subsequent telephone interviews during our study interval (1998–2008). The HRS permitted proxy respondents under some circumstances, but all proxy data were excluded from the analyses reported here.

Female cohort members were included in our study if they met three criteria. These included: (1) completing an interview in 1998 and that yielded usable alcohol data; (2) reporting their age as 50–65 years at the 1998 interview; and (3) completing at least two more interviews with usable alcohol data between 2000 and 2008. “Usable” data were defined as self-reported (non-proxy) responses with no missing values. Among the 5,752 women ages 50–65 at their 1998 interview, 4,439 (77.2%) completed at least two more interviews with useable alcohol data. Among the remaining 1,313 women, 329 (5.7% of the total sample) died before the 2008 interview, 823 (16.1%) were lost to follow-up for other reasons, and 153 (2.6%) had too many non-proxy interviews. The percent of study women who completed their biannual interview varied from a high of 87.8% in 2002 to a low of 77.8% in 2008.

### 2.2. Measurement

All 1998–2008 biannual interviews administered the same set of alcohol questions. Participants who responded affirmatively to *“Do you ever drink any alcoholic beverages such as beer, wine, or liquor?*” were asked three additional questions. “*In the last three months, on average, how many days per week have you had any alcohol to drink?*” “*In the last three months, on the days you drink, about how many drinks do you have?*” And “*In the last three months, on how many days have you had four or more drinks on one occasion?*” We used the number of drinks per day (zero for nondrinkers) in our trajectory models.

In other analyses, we considered drinks per week (drinking days per week x number of drinks per day), binge drinking (4+ drinks per occasion) and the 4-item CAGE [29] score. Like others [30,31], we classified women with CAGE scores of two or higher as having a history of problem drinking. The CAGE was administered once, typically during the initial HRS interview.

To identify other potential predictors (covariates), we reviewed the 1998 HRS questionnaire to identify data elements representing patient characteristics at baseline that could be readily assessed in primary care settings. The following variables were selected. Demographic data included age, race, ethnicity, education, and marital status. Health behaviors included tobacco use and whether or not vigorous exercise at least three times a week was reported. Health data included items on body mass index, self-reported health, problems with pain, depression, and chronic disease. Depression was defined as a score ≥ 5 on the 8-item CES-D scale [32]. Women were classified as having a chronic disease in 1998 if they reported that a doctor had ever told them they had hypertension, diabetes, cancer, chronic lung disease, congestive heart failure, stroke, or arthritis. Retirement status was obtained from HRS files prepared by RAND [33]. In 2008, the HRS added questions to assess menopausal status, but we found the data insufficient to reliably identify participants who were post-menopausal at baseline.

### 2.3. Analysis

To model intra-individual changes in the number of drinks per drinking day during the study interval, we derived semi-parametric group-based models [26,27]. All models included base-year (1998) sampling weights, adjusted for age in that year, and used log-transformed drinks per day values to limit skew. Quadratic and cubic terms were tested in all models. The SAS Proc Traj modeling algorithm includes cases with some missing data in the model as long as at least two non-missing observations are available for each case [25]. We required at least three alcohol assessments to enhance validity.

The best-fitting model was selected using three criteria. Bayesian Information Criterion (BIC) values, which increase as model fit improves, were used to compare models with “n” and “n−1” trajectories (e.g., 4 *versus* 3 distinct trajectory groups) [25,26]. Following convention, we interpreted 2*ΔBIC values of 6–10 as strong evidence and values greater than 10 as very strong evidence against the null (n−1) model [24,25]. Like others [22,34], we also required a minimum mean posterior probability of group assignment in all groups ≥ 0.70. High mean posterior probability values characterize models with relatively distinct groups. Proc Traj automatically assigns individuals to the trajectory group associated with their highest posterior probability. Finally, all of the trajectory groups had to contain at least 5% of the study sample.

To estimate the weighted percentages of women associated with each trajectory, we used the group assignments generated by the best-fitting model. Analysis of variance (ANOVA) models were then used to compare drinking behaviors across the trajectory groups at each of the six biannual interviews. Analyses based on probabilistic group assignments is appropriate when classification is highly certain, because errors in inference are likely to be small [24].

After conducting ANOVA and chi square comparisons to identify baseline characteristics that significantly distinguished the trajectory groups, we used adjusted logistic regression to identify risk factors associated with a substantial change in drinking over our 10-year interval including following an increasing (Model 1) or decreasing (Model 2) drinking trajectory from 1998 on. We did not compare long-term drinkers and non-drinkers in a logistic model, because our focus was on older women who changed their alcohol use behaviors over time. All characteristics that were significantly associated with drinking in the bivariate comparisons were forced simultaneously into the adjusted logistic regression models. Base-year sampling weights were also included in all models as recommended by the HRS documentation [28]. Adjusted odds ratios (AOR) with values above 1.0 and 95% confidence intervals (95%CI) that exclude the null identify factors that may increase the risk of following a trajectory that diverged from the 1998 value. AOR values below 1.0 with a 95%CI that excludes the null suggest factors that may reduce the risk of following the divergent trajectory.

## 3. Results and Discussion

### 3.1. Number of Distinct Trajectories and Weighted Proportions of Sample

**Figure 1.**
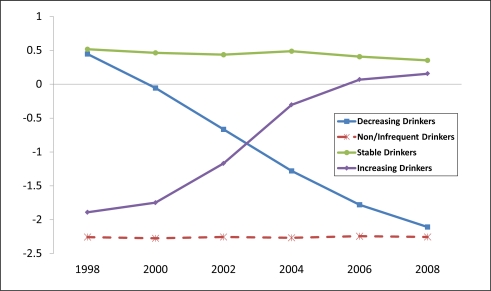
Alcohol use trajectories among 4,439 women in the HRS ages 50–65 years in 1998 based on number of drinks per drinking day (log scale).

The best-fitting model had four trajectories (Figure 1). All models with more than four trajectories failed to converge. The mean posterior probability of group assignment for all trajectories was high (≥0.94). We labeled the bottom curve (Figure 1, red line) as “*Non/Infrequent (‘Infrequent’) Drinkers*” because women in that group averaged less than 0.1 drink per day in all years.

In contrast, average intake reported by the “*Increasing Drinkers*” (purple line) climbed monotonically from 0.2 drinks per day in 1998 to 1.4 in 2008. The opposite pattern was observed among the *Decreasing Drinkers* (blue line) who reported intake levels that declined sharply from 1.8 drinks per day in 1998 to less than 0.1 in 2008. The *Stable Drinkers* (green line) reported about 1.8 drinks per day through 2004 and then decreased slightly to 1.6 drinks for 2006 and 2008. The model assigned 5.3% of the women to the *Increasing Drinkers* group, 5.9% to the *Decreasing Drinkers* group, 24.2% to the *Stable Drinkers*, and 64.6% to the *Infrequent Drinkers* group.

**Figure 2.**
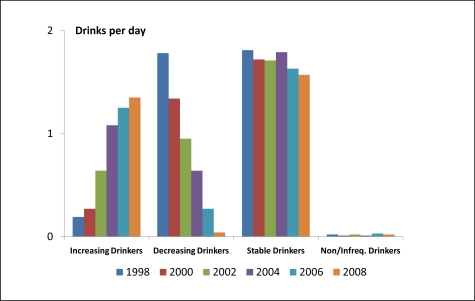
Numbers of drinks per drinking day by trajectory group and year among 4,439 women in the HRS ages 50–65 years in 1998.

**Figure 3.**
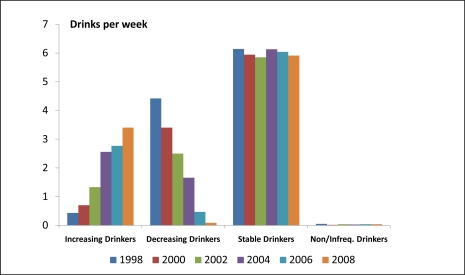
Numbers of drinks per week by trajectory group and year among 4,439 women in the HRS ages 50–65 years in 1998.

When ANOVA methods were used to compare the biannual alcohol use behaviors across these four groups of women, we found that the average numbers of drinks per drinking day and drinks per week differed significantly in all years (p < 0.0001, df = 3, F > 600 in all comparisons). Mean values (normal scale) for drinks per day and drinks per week for all assessment years are shown in Figures 2 and 3, respectively. Findings related to binge drinking were similar (data not shown). The percentage of *Increasing Drinkers* who reported binge drinking increased from 0.9% in 1998 to 9.2% in 2008. Corresponding values for the *Decreasing Drinkers* were 13.3% and 0.0%, respectively.

These findings are consistent with other research on alcohol use among older women suggesting some women drink moderately for many years, while others gradually reduce their use of alcohol. Previously, we derived 8-year drinking trajectories using National Longitudinal Survey (NLS) data from 1,658 women [12]. These women, all ages 50–65 in 1995, reported on their recent drinks per day during three or more biannual interviews completed between 1995 and 2003. The best-fitting NLS model also had four trajectories (*Increasing Drinkers*–8.8% of the sample, *Decreasing Drinkers*–8.6%, *Consistent Drinkers*–21.2%, and *Non/Infrequent Drinkers*–61.4%). The Wisconsin Longitudinal Study assessed past month drinking behaviors from 3,469 women at age 53 and then reassessed as many as possible at age 64 [35]. The percentage who were moderate drinkers remained constant at 56%, but the percentage who were non-drinkers increased slightly from 33% to 36% while the percentage of heavy drinkers declined from 11% to 9%. Similarly, a 10-year follow-up assessment on 529 U.S. women ages 55–65 at baseline found the proportion of drinking days per week dropped steadily over time, but many continued to drink several days per week [36].

It has been well-established for many years that women can become problem drinkers at any age and may develop alcohol-related problems more rapidly than age-comparable men. However, we believe our analyses are the first to demonstrate that a non-trivial percentage of older women increase their alcohol use over a long time frame. By 2008, the *Increasing Drinkers* were modestly exceeding the recommended limit of no more than one drink per day, although they averaged only 3.4 drinks per week, which is below the seven drinks per week limit.

The results reported here for older women differ in several respects from the findings that emerged during our parallel research on older men. Among HRS men ages 50–65 in 1998 who also completed at least three biannual interviews with valid alcohol use data in 1998–2008, the best-fitting trajectory based on reported numbers of drinks per day had 5 distinct trajectories. These trajectories were labeled as *Non/Infrequent Drinkers* (40.6% of the sample), *Increasing Drinkers* (5.5%), *Decreasing Drinkers* (7.6%), *Moderate Drinkers* (30.7%) and *At-risk Drinkers* (15.6%) [16]. The “at-risk” drinkers averaged more than 3 drinks per day during all years, while the *Moderate Drinkers* reported amounts similar to those observed among women in our *Stable Drinkers* group (1.6–1.8 drinks per day).

### 3.2. Risk Factor Analyses

Among the older women in the analyses reported here, chi-square and ANOVA comparisons of baseline characteristics across the four trajectory groups identified many statistically significant differences, although the magnitude of the differences was small in some instances (Table 1). Mean age ranged from a low of 57.6 years in the *Stable Drinkers* to a high of 58.4 years in the *Decreasing Drinkers* (p < 0.001). Much larger differences were observed for other variables. For example, the percentages who reported being frequently troubled by pain in 1998 ranged from a low of 19.8% among the *Stable Drinkers* to a high of 34.1% among the *Infrequent Drinkers* (p < 0.001). The drinking trajectory groups did not differ significantly on the mean number of interviews completed during our study interval. Overall, 68.6% of the women completed 5 or all 6 of the study interviews.

Using all 1998 demographic, health, and alcohol/tobacco use characteristics in Table 1 and the base-year sampling weights, we constructed two adjusted logistic regression models. Model 1 compared the 232 *Increasing Drinkers* (purple line in Figure 1) who averaged 0.2 drinks per day in 1998 to the 2,867 *Infrequent Drinkers* (red line) who averaged <0.1 drinks per day. On a multiplicative scale, this difference is notable, but the absolute difference is small. Model 2 compared the 264 *Decreasing Drinkers* (blue line) to the 1,076 *Stable Drinkers* (green line). Both of these groups averaged 1.8 drinks per day in 1998.

Model 1 results (Table 2) suggest infrequent drinkers in 1998 who increased the amount of alcohol consumed over the next 10 years were more likely than those who continued as non/infrequent drinkers to have more education (AOR = 1.59, 95%CI: 1.19–2.11), engage in vigorous physical activity (1.41, 1.06–1.86), and report that they had recently consumed 4 or more drinks on one occasion (4.34, 1.07–17.59). However, it is important to note that at baseline, binging was rarely reported in either group (*Increasing drinkers*: 0.9%, *Infrequent Drinkers*: 0.2%). The *Increasing Drinkers* were less likely to be obese in 1998 (0.59, 0.42–0.84) or report current smoking (0.67, 0.47–0.94).

Women who reported drinking more than one drink per day in 1998 but then steadily decreased their intake during follow-up were more likely than those who continued as stable drinkers (Table 2, Model 2) to report depression (AOR = 2.16, 95%CI: 1.29–3.61) and cigarette smoking (1.50, 1.05–2.15). Problem drinking is well-established as a risk factor for depression and depressed individuals are routinely advised to limit their intake of alcohol [37]. They were less likely to be retired (0.63, 0.41–0.98) or report recently drinking 4 or more drinks on one occasion (0.40, 0.25–0.61).

The results observed for education, retirement, and recent binge drinking were expected, but the magnitude of the AORs associated with consuming four or more drinks on one occasion was striking. Infrequent drinkers at baseline who reported drinking this heavily at least once in the last three months were four times more likely to report higher levels of drinking on subsequent interviews. Conversely, stable drinkers who reported any binge drinking were far less likely to sharply reduce their alcohol intake over follow-up. Because recent binging at baseline was positively associated with a CAGE score ≥ 2 (X^2^ = 138.6, p < 0.0001), we expect that a few *Increasing Drinkers* who were abstinent at baseline may be women with a history of alcohol problems who resumed drinking after the 1998 interview.

The finding that *Increasing Drinkers* were more likely to report vigorous exercise in 1998 is consistent with a small body of recent studies showing a strong positive, but not necessarily causal, correlation between physical activity and alcohol use [38]. The observations that *Increasing Drinkers* were more likely to report exercise, less likely to be obese, and less likely to smoke may all reflect an underlying association between health behavior or lifestyle and alcohol use. The associations are not paradoxical, because many older women know that moderate alcohol use provides important health benefits and may choose to drink for that reason. The adjusted odds ratios for these variables in Model 2, although not statistically significant, were also consistent with a positive correlation between other health behaviors and drinking. A cross-sectional study of alcohol use among 6,917 Swedish women ages 50–59 similarly found that nondrinkers had poorer health and more physical symptoms than weekly drinkers [39]. Others have also found a faster decline in alcohol consumption among older adults who smoke [40].

We searched the literature for comparable longitudinally-based, alcohol-related risk factor analyses specific to older women and were unable to find appropriate comparison studies. Adjusted logistic regression models were not derived for the NLS data mentioned above due to the smaller size of the sample (1,658 women *versus* the 4,439 in the HRS) and the termination of NLS data collection in 2003. In our analyses of risk factors among HRS men, *Increasing Drinkers* were also more likely to report vigorous exercise and less likely to report smoking. *Decreasing Drinkers* were also less likely to report binge drinking [16]. Other results were dissimiliar.

## 4. Conclusions

Findings from this study are likely to be valid and generalizable to other older women in the U.S. for several reasons. All study participants were randomly sampled women who completed an identical series of alcohol questions at least three times over a 10-year interval. Sample weights were included in all analyses. The trajectory models controlled for age in 1998 and the mean posterior probabilities of group assignments were high (≥0.94).

However, several limitations deserve mention. Some ethnic and racial minority groups were under-represented, and accuracy of the self-reported data could not be ascertained. Other limitations of the alcohol data include the absence of “standard” drink references and the untested assumption that drinking during the last three months typifies drinking since the last biannual interview. Study findings have important implications for public health practice and clinical medicine. First, nearly one-third (30.2%) of all study women ages 50–65 in 1998 were averaging more than one drink per day at baseline and thus exceeding current guidelines. Most of these women continued to drink at close to that level for the next 10 years. Older women who average more than one drink per day are more likely to gain excess weight, experience adverse side effects from many commonly prescribed medications, develop other alcohol-related problems, and may die at an earlier age. However, a similar proportion (29.5%) were essentially abstinent at baseline and thus deprived of the potential health benefits of moderate drinking. It is now well-established that drinking no more than one drink per day or seven drinks per week may provide cardiovascular and cognitive benefits to postmenopausal women and also lower all-cause mortality.

About 5.3% of the women abstinent at baseline increased their intake through at least the 2008 interviews and were modestly exceeding the one drink per day limit by 2004, while 5.9% of those drinking regularly in 1998 were abstinent by 2008. Although the adjusted logistic regression models identified some characteristics that may predict a change in long-term drinking, our findings suggest that it will be difficult to identify women likely to increase or decrease their drinking in advance of any such change. Many older female patients report that they have more than 12 years of education and exercise frequently. However, only a relatively small subset of these patients are likely to markedly increase their drinking over the next 10 years. Taken together, the evidence suggests an on-going need for public health education on both the risks and benefits of drinking among older women. The data also underscore the importance of including questions about recent alcohol use during routine (non-urgent) patient encounters, including those with women who previously reported non-drinking. The research reported here also has implications for future research on alcohol use among women ages 50 and older. Our finding that 11.2% of all older women (the *Increasing Drinkers* and the *Decreasing Drinkers*) are likely to markedly change their level of alcohol intake over a 10-year interval suggests the need for substantial caution when interpreting observations based on single (cross-sectional) assessments.

## Figures and Tables

**Table 1.** Characteristics of alcohol use trajectory groups including study participation and demographic, health, and alcohol/tobacco use history at baseline (1998) among 4,439 women in the Health and Retirement Study. ^†^

	**Increasing Drinkers**	**Decreasing Drinkers**	**Stable Drinkers**	**Infrequent Drinkers**
**Number (%) in group**	232 (5.3)	264 (5.9)	1,076 (24.2)	2,867 (64.6)

**Trajectory characteristics**				

Posterior probability	0.95	0.94	0.99	0.99
# of interviews completed (s.d.)	4.3 (1.1)	4.2 (1.0)	5.0 (1.1)	5.1 (1.1)
# (%) known/presumed alive in 2008***	229 (98.7)	245 (92.8)	1,048 (97.4)	2,654 (92.6)

**Demographic characteristics**				

Age (s.d.)***	57.7 (4.2)	58.4 (4.5)	57.6 (4.3)	58.3 (4.2)
Race***				
% Black	15.9	17.4	8.0	22.0
% White	80.2	80.7	90.5	72.4
% “other”	3.9	1.9	1.5	5.6
% Hispanic***	9.9	8.7	3.1	11.4
% with >12 years of education***	40.9	40.9	55.8	28.9
% married or cohabiting***	72.8	70.5	76.2	67.4
Retirement status***				
% not retired	56.5	54.9	60.3	46.6
% partially retired	8.2	8.7	10.6	8.2
% retired	12.9	20.8	14.8	17.7
% other	22.4	15.5	14.3	27.5

**Health status**				

% health as excellent/very good***	47.8	43.2	63.9	34.6
% with ≥1 chronic disease (see text)***	67.7	77.6	64.8	77.4
% reported vigorous physical activity***	50.4	48.1	54.4	41.8
%with depression (CES-D score ≥5)***	12.1	14.4	5.4	15.4
% frequently troubled by pain***	26.7	32.6	19.8	34.1
% with body mass index ≥30 (obese)***	24.1	23.1	14.7	33.8

**Alcohol and tobacco at baseline**				

% regular smoker (cigarettes)*	17.4	28.2	22.8	21.6
% with ≥4 drinks on one occasion***	0.9	13.3	22.2	0.2
% with alcohol CAGE score ≥2***	9.5	8.7	10.6	6.0

† Chi square and ANOVA testing used to assess statistical significance across all trajectory groups;

* p < 0.05;

** p < 0.01;

*** p < 0.001.

**Table 2.** Adjusted odds ratios and 95% confidence intervals for 1998 characteristics and changing *versus* consistent drinking trajectories among HRS women ages 50–65.^†^

	**Increasing Drinkers *vs.* Non/Infrequent Drinkers Model 1**	**Decreasing Drinkers *vs.* Stable Drinkers Model 2**
**Age**		

60 years and older	0.81 (0.59–1.10)	1.01(0.71–1.42)
50–59 years	1.00	1.00

**Race**		

Black	0.79 (0.49–1.26)	1.71 (0.98–2.98)
Other	0.70 (0.34–1.42)	0.64 (0.21–1.96)
White	1.00	1.00

**Hispanic ethnicity**		

Hispanic	1.03 (0.61–1.75)	1.89 (0.88–4.03)
Non-Hispanic	1.00	1.00

**Education**		

>12 years	1.59 (1.19–2.11)**	0.91 (0.67–1.25)
12 years or less	1.00	1.00

**Marital status**		

Married or cohabiting	1.07 (0.78–1.45)	0.74 (0.52–1.04)
Not married or cohabiting	1.00	1.00

**Retirement status**		

Not retired	1.53 (0.97–2.40)	0.63 (0.41–0.98)*
Partially retired	1.35 (0.71–2.57)	0.64 (0.35–1.17)
Other/not relevant	1.39 (0.85–2.27)	0.79 (0.47–1.35)
Fully retired	1.00	1.00

**Self-reported health**		

Excellent or very good	1.23 (0.89–1.71)	0.75 (0.53–1.07)
Good, fair, or poor	1.00	1.00

**Chronic disease reported in 1998**		

Yes	0.82 (0.60–1.12)	1.36 (0.95–1.93)
No	1.00	1.00

**Reports vigorous physical activity**		

Yes	1.41 (1.06–1.86)*	0.85 (0.63–1.15)
No	1.00	1.00

**Depression**		

Yes (8-item CES-D ≥ 4)	0.85 (0.53–1.36)	2.16 (1.29–3.61)**
No	1.00	1.00

**Frequently troubled by pain**		

Yes	0.91 (0.65–1.29)	1.24 (0.86–1.79)
No	1.00	1.00

**Body mass index**		

Obese (BMI ≥ 30.0)	0.59 (0.42–0.84)**	1.25 (0.84–1.86)
Not obese (BMI < 30.0)	1.00	1.00

**Current cigarette smoker**		

Yes	0.67 (0.47–0.94)*	1.50 (1.05–2.15)*
No	1.00	1.00

**≥4 drinks on one occasion (binging)**		

Yes	4.34 (1.07–17.59)*	0.40 (0.25–0.61)***
No	1.00	1.00

**Alcohol CAGE score ≥2**		

Yes	1.23 (0.75–2.02)	0.83 (0.49–1.40)
No	1.00	1.00

† All terms forced simultaneously into models that included base-year sampling weights.

* p < 0.05;

** p < 0.01;

*** p < 0.001.
